# Linking inherent O-Linked Protein Glycosylation of YghJ to Increased Antigen Potential

**DOI:** 10.3389/fcimb.2021.705468

**Published:** 2021-08-19

**Authors:** Mette Thorsing, Thøger Jensen Krogh, Lars Vitved, Arkadiusz Nawrocki, Rikke Jakobsen, Martin R. Larsen, Subhra Chakraborty, A. Louis Bourgeois, Ann Zahle Andersen, Anders Boysen

**Affiliations:** ^1^GlyProVac LLC, Odense, Denmark; ^2^Department of Cancer and Inflammation Research, University of Southern Denmark, Odense, Denmark; ^3^Department of Biochemistry and Molecular Biology, University of Southern Denmark, Odense, Denmark; ^4^Center for Immunization Research, Johns Hopkins Bloomberg School of Public Health, Baltimore, MD, United States; ^5^Center for Vaccine Innovation and Access, PATH, Washington, DC, United States

**Keywords:** Enterotoxigenic *Escherichia coli*, immunogenicity, vaccine development, mass spectrometry, protein glycosylation, sub-unit vaccines, YghJ, SslE

## Abstract

Enterotoxigenic Escherichia coli (ETEC) is a WHO priority pathogen and vaccine target which causes infections in low-income and middle-income countries, travelers visiting endemic regions. The global urgent demand for an effective preventive intervention has become more pressing as ETEC strains have become increasingly multiple antibiotic resistant. However, the vaccine development pipeline has been slow to address this urgent need. To date, vaccine development has focused mainly on canonical antigens such as colonization factors and expressed toxins but due to genomic plasticity of this enteric pathogen, it has proven difficult to develop effective vaccines. In this study, we investigated the highly conserved non-canonical vaccine candidate YghJ/SsLE. Using the mass spectrometry-based method BEMAP, we demonstrate that YghJ is hyperglycosylated in ETEC and identify 54 O-linked Set/Thr residues within the 1519 amino acid primary sequence. The glycosylation sites are evenly distributed throughout the sequence and do not appear to affect the folding of the overall protein structure. Although the glycosylation sites only constitute a minor subpopulation of the available epitopes, we observed a notable difference in the immunogenicity of the glycosylated YghJ and the non-glycosylated protein variant. We can demonstrate by ELISA that serum from patients enrolled in an ETEC H10407 controlled infection study are significantly more reactive with glycosylated YghJ compared to the non-glycosylated variant. This study provides an important link between O-linked glycosylation and the relative immunogenicity of bacterial proteins and further highlights the importance of this observation in considering ETEC proteins for inclusion in future broad coverage subunit vaccine candidates.

## Introduction

Whereas an increase in the availability of resources such as clean water and professional healthcare has significantly decreased the mortality from *E. coli* infections in low and middle-income countries (LMICs), the number of non-fatal infections remains high and so does the cost of these infections to societies in low-resource settings due to childhood stunting and delays in cognitive development as well as increased risk of dying from other infectious diseases (Anderson et al., 2019; Khalil et al., 2021). In addition, *enterotoxigenic Escherichia coli* (ETEC) globally continues to cause severe diarrhea and death in high risk patient groups, including older individuals (Poolman and Anderson, 2018) and is the most common cause of diarrhea in travelers to endemic regions (Olson et al., 2019).

*E. coli* remains a World Health Organization (WHO) priority pathogen and vaccine target given its high burden and the increasing emergence of Extended-spectrum β-lactamase producing Enterobacteriaeceae including ESBL-ETEC (Shrivastava et al., 2018; Tacconelli et al., 2018). In a recent report, the Wellcome Trust and Boston Consulting Group recommend that vaccine development for enteric *E. coli* including ETEC be accelerated due to the increasing antimicrobial resistance (AMR) threat (Wellcome Trust, 2019) and this recommendation was repeated in the WHO Action Framework: Leveraging Vaccines to Reduce Antibiotic Use and Prevent Antimicrobial Resistance (World Health Organization, 2020). In striking contrast to the increasing need for therapeutic interventions, the *E. coli* vaccine pipeline is limited to only 16 vaccine candidates, ten of which are in the research/preclinical phase. Thus, whereas ETEC is a global challenge, both the commercial and academic *E. coli* vaccine pipeline remains inadequate (Barry et al., 2019; Theuretzbacher et al., 2019; Bekeredjian-Ding et al., 2020; Giersing, 2020).

The traditional canonical antigens of ETEC include colonization factors and secreted toxins. Past efforts in *E. coli* vaccine development have focused mainly on these important virulence factors. However, *E. coli* displays huge genomic plasticity, resulting in large variations in virulence factors with each pathotype within the species, which hinders the development of a vaccine with broad coverage based on these canonical antigens (Turner et al., 2006; Moriel et al., 2012; Nesta and Pizza, 2018). With an increased understanding of the complexity of *E. coli* pathogenesis, significant efforts have been devoted to the discovery and characterization of novel non-canonical antigens (Roy et al., 2010; Fleckenstein et al., 2014; Chakraborty et al., 2016). These antigens form a group of molecular entities identified to be relevant for either pathogenesis, immunology or vaccinology.

One of the non-canonical antigens, which has received significant attention, is YghJ, also known as SslE (Nesta et al., 2014; Chakraborty et al., 2018). YghJ is a secreted and broadly conserved metalloprotease within the pathogenic *E. coli* family (Luo et al., 2014). During the early stages of infection, YghJ degrades the protective intestinal mucin layer, facilitating access to the epithelial cell surface and colonization, as well as toxin delivery. Moreover, proteomic and transcriptomic analyses show that YghJ is immunogenic in both animals and humans and that expression increases upon adherence to host cells (Roy et al., 2010; Kansal et al., 2013; Chakraborty et al., 2018). From the host’s perspective, it has been demonstrated that YghJ from *E. coli* strains associated with neonatal sepsis not only causes *in vitro* stimulation of proinflammatory cytokines in a human intestinal epithelial cell line, but also induces damage to mouse ileal tissues *in vivo* (Tapader et al., 2016; Tapader et al., 2017). Lastly, immunization with YghJ has been shown to confer some protection in animals against extraintestinal pathogenic *E. coli* bacteremia (Moriel et al., 2010), uropathogenic *E. coli* pyelonephritis, ETEC colonization of caecum and *E. coli* caused sepsis (Nesta et al., 2014). In addition to academic interest, YghJ has also been pursued commercially by Novartis and later Glaxo Smith Kline (Moriel et al., 2010; Serino et al., 2010; Nesta et al., 2014). However, despite the continuous attention on YghJ in ETEC controlled human infection model (CHIM) studies and the established role of YghJ as an important factor in effective intestinal colonization (Fleckenstein et al., 2014; Chakraborty et al., 2018; Mirhoseini et al., 2018; Nesta and Pizza, 2018; Vedøy et al., 2018), YghJ has, to the best of our knowledge, not progressed as a vaccine candidate antigen beyond early animal challenge studies.

With the discovery and identification of O-linked glycosylated proteins, an extra layer of complexity has been added to bacterial pathogenesis. O-linked protein glycosylation, the addition of glycans to either Serine (Ser) or Threonine (Thr) amino acid residues, is well documented in diverse Gram-negative bacterial species such as *Neisseria gonorrhoeae*, *Burkholderia cenocepacia*, *Acinetobacter baumannii*, *Pseudomonas aeruginosa* as well as *E. coli* (Benz and Schmidt, 2001; Castric et al., 2001; Vik et al., 2009; Iwashkiw et al., 2012; Lithgow et al., 2014). The species-specific glycans used for protein glycosylation are remarkably diverse but nevertheless important as their loss results in reduced virulence potential, reduced fitness and altered biophysical properties of e.g. adhesins (Knudsen et al., 2008; Faulds-Pain et al., 2014; Schäffer and Messner, 2017; Mohamed et al., 2019). Furthermore, protein glycosylation also appear to increase the antigenic variation in order to evade the immune system of the host (Gault et al., 2015). The most comprehensive O-linked protein glycosylation studies include high-throughput mass spectrometry and the dedicated glycoproteomics technique termed BEMAP (β-elimination of O-linked carbohydrate modifications, Michael addition of 2-Aminoethyl phosphonic acid) (Boysen et al., 2016; Scott, 2019). The BEMAP technique (patent US 10,647,749 B2) can be employed to map O-linked glycoproteins from any biological source and was developed with the intention to identify and expand the repertoire of glycosylated proteins linked to ETEC pathophysiology. Using BEMAP, more than 140 glycoproteins associated with the outer membrane fraction and outer membrane vesicles were previously identified in *E. coli* K-12 and ETEC H10407 and a potential link between pathogenesis and O-linked glycosylation was discussed (Boysen et al., 2016). This was based on the remarkable finding that protein glycosylations were only found in the pathogenic ETEC strain despite that most of the identified glyco-proteins were conserved on a protein level between ETEC and the commensal *E. coli* K-12. In addition, it was observed that the majority of canonical as well as non-canonical ETEC virulence factors were glycosylated, including YghJ.

In the present study we have further investigated the extent of YghJ glycosylation and coupled this inherent O-linked protein glycosylation to an increased antigenic potential of YghJ. We have expressed and purified glycosylated YghJ from the canonical ETEC strain H10407 and performed an in depth BEMAP analysis to identify glycosylated Ser/Thr residues. Using BEMAP, we identified 54 modified residues within this 1519 amino acid protein. To obtain a control protein for the investigation of the potential impact of O-linked glycosylation on protein immunogenicity we over-expressed and purified a non-glycosylated version of YghJ. This control antigen was overexpressed from a K-12 MG1655Δ*hldE* genetic background and the absence of O-linked glycosylation was confirmed using BEMAP. In pathogenic *E. coli*, HldE catalyzes the biosynthesis of ADP-activated heptose precursor units which are used in protein glycosylation (Benz and Schmidt, 2001; Nakao et al., 2012). Therefore, with the deletion of hldE, the expression strain loses its ability to add heptose glycans to YghJ.

To examine the difference in immune response towards glycosylated YghJ and the non-modified protein variant subsequent to ETEC infection, serum isolated from pre- and post H10407 challenged volunteers was used in ELISA experiments (Chakraborty et al., 2016). Both glycosylated YghJ and non-glycosylated YghJ were recognized by sera from patients prior to infection (day 0). Importantly, the increase in recognition of glycosylated YghJ from days 0 to day 7 and 28 post infection was significantly greater than recognition of non-modified YghJ variant at both time points.

The current study shows that the bacterial mucinase YghJ is a hyper O-glycosylated protein. We also show that antibodies from patients exposed to ETEC infections predominantly recognize glycosylated over non-glycosylated YghJ which points to increased immunogenicity of glycosylated YghJ compared to the non-glycosylated antigen. The current study therefore highlights the importance of considering O-linked glycosylation when considering the role of bacterial proteins in pathogenic evolution but also from the perspective of antigen discovery and the development of effective and more broadly protective vaccines.

## Materials and Methods

### Bacterial Strains and Culture Conditions

Strains were grown in Luria Bertani (LB) (Sambrook and Green, 2012) or M9 minimal medium (Boysen et al., 2010) supplemented with 0.4% glucose and 0.2% casamino acids. Cells used for electroporation were grown in Super Optimal Broth (SOB) and Super Optimal Broth with Catabolite repression (SOC) (Hanahan, 1983). Protein expression was induced from the P_A1/04/03_ promoter by 1 mM isopropyl-β-d-thiogalactopyranoside (IPTG). When required, the media was supplemented with either 40 µg/ml Kanamycin or 30 µg/ml Chloramphenicol. Strains and plasmids are listed in **Supplementary Table S1** and primers are listed in **Supplementary Table S2**.

### DNA Manipulations

The Datsenko and Wanner system and primers JMJ388 and JMJ389 were used to delete *hldE* in MG1655 (Datsenko and Wanner, 2000). The *hldE* gene plays a role in the biosynthesis of ADP-activated heptose units required for post-translational protein heptosylation (Benz and Schmidt, 2001; Nakao et al., 2012). Candidate clones were selected, isolated and tested by PCR using the primer pairs JMJ390+JMJ391 and JMJ390+JMJ99.

To isolate glycosylated YghJ, a 3xFLAG epitope tag was added to the *yghJ* gene on the ETEC H10407 chromosome as described by Uzzau et al. (2001). In brief, a PCR product generated using pSUB11 as template and the primer pairs GPV18+GPV19 was electroporated into *E. coli* H10407. Transformants were selected on LB agar plates containing 40 µg/ml kanamycin. The primer pairs GPV16+GPV17 as well as GPV67+GPV147 were used to verify H10407*yghJ* 3xFLAG. The construct was verified by sequencing.

To isolate a non-glycosylated YghJ protein version, a 3xFLAG epitope tag was added to the *yghJ* gene and put under an IPTG inducible promoter for expression in a MG1655Δ*hldE* mutant strain background. Briefly described, An IPTG inducible promoter and the 3xFLAG epitope was added to the *yghJ* gene in two steps. First, the primers GPV95 and GPV97 were used to generate a PCR product using chromosomal DNA from the ETEC H10407*yghJ* 3xFLAG strain as template. In the second step, GPV96 and GPV97 were used to generate a PCR product that was digested with XhoI and XbaI and subsequently ligated into pXG-0. This generated pGPV104. pGPV104 was verified by sequencing before transformation into the MG1655Δ*hldE* mutant strain background.

### Protein Purification

Glycosylated YghJ 3xFLAG was isolated from the ETEC H10407*yghJ* strain grown in M9 minimal medium supplemented with 0.2% glucose and 0.4% casamino acid and 40 μg/ml kanamycin. The culture was grown to OD_600 =_ 2.5 at 37°C after which it was harvested. The culture supernatant was sterile filtered (0.22μm pore size), NaCl and Triton X-100 was added to obtain a final concentration of 200 mM and 0.01%, respectively. Anti-FLAG M2 affinity agarose gel beads (SigmaAldrich; A2220) was used to capture the 3xFLAG epitope. Isolated FLAG affinity agarose beads were washed twice with FLAG Sup wash buffer I (400 mM NaCl, 0.1% Triton X-100, 1 mM EDTA in PBS buffer pH 7.6) and once with FLAG Sup buffer II (400 mM NaCl, 0.01% Triton X-100, 1 mM EDTA in PBS buffer pH 7.6). Elution was accomplished with Elution buffer (500 mM Arginine, 500 mM NaCl, pH = 3.5). Eluate fractions were spin filter concentrated before dialyzed against PBS at 4°C over night in a cold room.

Non-glycosylated YghJ 3xFLAG was isolated from the MG1655Δ*hldE*/pGPV104 strain grown in LB medium supplemented with 40 μg/ml Chloramphenicol. The culture was grown to OD_600 =_ 2.5 at 37°C after which it was harvested by centrifugation. Cell pellets were collected and 500 μg DNaseI was added before the sample was lysed three times in a French Press at 2.2 kbar. The lysate was cleared by ultracentrifugation at 125.000 x g at 4°C for three hours in a Beckman SW 32 Ti rotor. Sample volume was increased to 1 L and NaCl, Triton X-100 and EDTA was added to obtain a final concentration of 600 mM, 0.01% and 1 mM, respectively. Anti-FLAG M2 affinity agarose gel beads was added to the supernatant and incubated with shake at 4°C O/N in a cold room. FLAG affinity agarose beads were isolated, washed, eluted and dialyzed as described above.

### BEMAP and Mass Spectrometry Assisted Identification of Glycosylated Ser/Thr Residues

The BEMAP analysis was carried out as previously described (Boysen et al., 2016). A total of 40 µg protein was used as input to identify glycosylated YghJ Ser/Thr residues. As described in detail in (Boysen et al., 2016), raw data was generated on LTQ Orbitrap Velos, Orbitrap Velos Pro or Q-Exactive Plus mass spectrometers (Thermo Fisher Scientific, Bremen, Germany). Data was processed with Proteome Discoverer (Version 1.4.1.14, Thermo Fisher Scientific) and subjected to database searching using an in-house Mascot server (Version 2.2.04, Matrix Science Ltd., London, UK). Database searches were performed as previously described (Boysen et al., 2016). The mass spectrometry proteomics data have been deposited to the ProteomeXchange Consortium via the PRIDE (Perez-Riverol et al., 2019) partner repository with the dataset identifier PXD025876.

### Experimental Human Challenge Study

In a dose descending experimental challenge model, healthy American adult volunteers were challenged with an ETEC strain H10407 in 3 cohorts in an in-patient unit at Johns Hopkins University as described before (Chakraborty et al., 2016). Samples from cohort 2, where volunteers were challenged with 10^7^ CFU of ETEC, were used in this study. Subjects were excluded if they had significant medical problems; if an HIV-1, hepatitis B, or hepatitis C test was positive; or if they had traveled to countries where ETEC or cholera infection is endemic within two years prior to receipt of investigational agent. After challenge, subjects were monitored for signs and symptoms of enteric illness. All the subjects were treated with antibiotics 120 hours (5 days) after challenge, or earlier if required because of the diarrhea illness according to the clinical protocol. Diarrhea was classified as mild (1 to 3 diarrheal stools totaling 200 to 400 g/24 h), moderate (4 to 5 diarrheal stools or 401 to 800 g/24 h), or severe (6 or more diarrheal stools or ≥800 g/24 h). No diarrhea was defined as no loose stool observed. The volunteers challenged with 2x10^7^ dose resulted in attack rate of 67%.

### ELISA Experiment

96-well Maxisorb ELISA plates (Nunc, Denmark) were coated overnight at 4°C with glycosylated and non-glycosylated YghJ (2.5 μg/mL) in phosphate buffered saline (PBS). Plates were washed twice and blocked with PBS containing 0.05% Tween 20 for 15 min before the human serum samples (PBS containing 0.05% Tween 20 and 3% skimmed milk), from the experimental challenge model described above, were added to the plate. Each serum sample was two-fold serial diluted in 11 wells starting with a dilution factor of 25. The twelfth well was used for background determination (no serum). All three sera from each patient were analyzed on the same plate. After 1 hr of incubation with serum, the plates were washed 3 times with phosphate buffered saline (PBS) containing 0.05% Tween 20. Secondary polyclonal rabbit anti-Human IgG/A/M (DAKO P0212) HRP conjugated antibody was diluted x1000 and added to all wells in the plates. The plates were washed as described above before the signal strength was detected using TMB X-tnd (Kementec cat. no. 5280) and a Molecular Devices microplate reader set at 450nm. SoftMax Pro 7 software was used to fit measured intensity values (arbitrary units) as a function of serum concentration. Endpoint titers at 0.4 units above background were read from the fitted curve as described in Chakraborty et al., 2019 (Chakraborty et al., 2019).

### SDS-PAGE and Western Blots

Denaturing Western blotting was used to detect YghJ protein secreted to the culture supernatant as well as for the analysis of purified glycosylated YghJ and non-glycosylated YghJ. YghJ protein samples were boiled and run on PAGE gels as described in (Boysen et al., 2010). After the transfer, the membrane was blocked with 1% skimmed milk in PBS buffer with 0.05% Tween-20 for 1 hour. When analyzing human serum samples, the membrane was blocked with 3% skimmed milk in PBS buffer with 0.05% Tween-20 for 1 hour. Both primary and secondary antibodies were diluted into 1% skimmed milk in PBS buffer with 0.05% Tween-20. Incubation times were 1 hour. The antibodies were diluted as shown in **Supplementary Table S3**. Blots were developed using Immobilon Forte Western HRP substrate (Millipore). The signal was detected using an Amersham Imager 680 (Cytivalifesciences).

Native Western blotting was used to detect purified glycosylated YghJ and non-glycosylated YghJ. Briefly described, YghJ protein was mixed 1x SDS native loading buffer (60 mM Tris-HCl, pH 6.8, 10% glycerol, 0.005% bromphenol blue) at room temperature before loading onto a NUPAGE 4-12% Bis-Tris Gels (Invitrogen). Proteins were separated in a native MES buffer (50mM MES, 50mM Tris Base, 0.01% SDS, pH 7.3) after which they were transferred to a PVDF membrane as described above. The YghJ specific signal was obtained as described above.

### Statistical Analysis

For preparation of graphs and statistical analysis, we used Prism, version 6.07 (GraphPad Software, San Diego, CA). To test for differences in YghJ specific antibody levels in serum samples, between day 0 and day 7 as well as day 0 and day 28, we used Wilcoxon signed rank test. Minimum p-values, for which the null hypothesis was rejected, were reported. P-values ≤ 0.05 were considered significant.

## Results

### BEMAP Reveals Extensive YghJ O-Linked Protein Glycosylation

We have previously established a catalogue of O-linked glycosylated proteins in ETEC strain H10407 (Boysen et al., 2016; Maigaard Hermansen et al., 2018). More than 200 proteins were found to be modified and >800 specific glycosylated Ser/Thr residues were identified in the screening. Among these, the non-canonical antigen YghJ was found to be modified at four sites (Boysen et al., 2016). In the current study YghJ was affinity purified using a 3xFLAG epitope tag added to the C-terminus of the protein (Uzzau et al., 2001). Under standard laboratory conditions, ETEC secretes enzymatically active YghJ into the growth medium (Luo et al., 2014), thus, to ensure that only fully processed and modified YghJ was analyzed by BEMAP, we purified the exported protein from the culture supernatant. With an input of 40 μg purified YghJ, 28 peptides which contained a total of 54 glycosylated residues were identified using the BEMAP protocol, see **Table 1**.

**Table 1:** BEMAP analysis of glycosylated YghJ. YghJ was digested with Trypsin and O-linked glycosylated peptide sequences were identified using BEMAP in combination with mass spectrometry.

start	end	Sequence	Site#	Site#	Site#	Site#	Site#	Site#	Site#
91	101	TGYLTLGGsQR	99						
102	143	VtGAtCNGEssDGFtFKPGEDVtCVAGNtTIATFNTQSEAAR	103	106	111	112	116	124	130
169	199	SNAVSLVTsSNsCPANtEQVCLtFSSVIESK	177	180	185	191			
218	231	LVNEEVENNAAtDK	229						
355	364	YsttGQNNtR	356	357	358	363			
419	435	EIDtAICAKTDGCNEAR	422						
428	442	tDGCNEARWFsLttR	428	438	440	441			
456	468	LWGVDTNYKSVsK	467						
469	486	FHVFHDStNFYGsTGNAR	476	481					
487	504	GQAVVNIsNAAFPILMAR	494						
588	609	DGQCtLNsDPDDMKNFMENVLR	592	595					
610	615	YLsnDR	612						
622	636	ssMtVGTNLEtVYFK	622	623	625	632			
720	734	GGsVLIMENVMSNLK	722						
735	743	EEsAsGFVR	739						
757	769	sVVnNDPQGYPDR	757						
818	830	LEVAsWQEEVEGK	822						
845	853	TPEsLAAAK	848						
909	922	AMLQAADLGtNIQR	918						
923	935	LYQHELYFRtNGR	932						
1000	1015	KsLIDNKMIYGEEssK	1001	1013	1014				
1016	1034	AGMMNPsYPLNYMEKPLTR	1022						
1048	1075	VDVEKYPGVVNtNGEtVtQNINLYSAPTK	1059	1063	1065				
1101	1120	StVPVtVTVALADDLtGREK	1102	1106	1116				
1134	1142	tYDLKANDK	1134						
1134	1146	TYDLKANDKVtFK	1144						
1341	1362	VADDITVAPEYLEEsNGQAWAR	1355						
1418	1431	ARGDEVsNDKFGGK	1424						

The start and end position of each of the 28 peptide sequences within YghJ are listed, as well as the specific residue number which is modified. Lower case s or t indicate modified residue.

Thus, whereas the previous screening revealed four glycosylated sites in YghJ, our in depth analysis shows that approximately 25% of all the Ser/Thr residues in YghJ are O-linked glycosylated. When assigning the glycosylation site to the primary sequence of YghJ, the modifications were more or less evenly distributed throughout the protein. We next investigated if the O-glycosylation sites were randomly distributed within the protein structure or if they clustered into particular spatial regions, which could be of biological importance. Unfortunately, an YghJ crystal structure remains to be resolved. However, we used the Protein Homology/analogY Recognition Engine (Phyre2) to generate a 3D model of the protein. The algorithm was able to assign a structure to the last 500 aa of the protein and we visualized the structural position of the O-glycosylation sites by highlighting sugar-modified residues within the structures, see **Supplementary Figure S1**. We found that in general, the glycosylation sites were surface exposed and located in unstructured regions on the protein. In previous studies it has been speculated that the spatial arrangement of the glycosylated residues could be a mode to scramble the surface structure in order to evade recognition by the immune system (Gault et al., 2015). Based on the Phyre2 model prediction and the location of the glycans, it is possible that the YghJ modifications serve the same immunologic purpose.

### Purification and Characterization of Glycosylated and Non-Glycosylated YghJ

**Figure 1:**
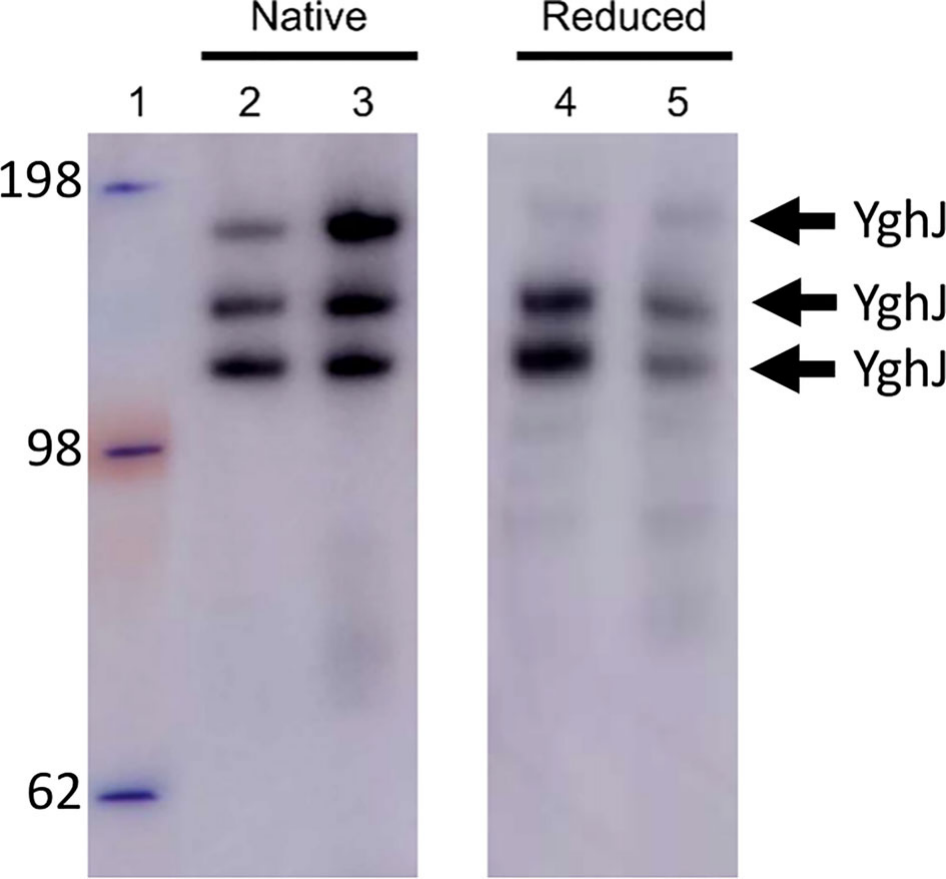
Western blot analysis of purified glycosylated and non-glycosylated YghJ. Glycosylated and non-glycosylated YghJ was loaded onto a PAGE gel and run under either non-reducing (left panel) or reducing conditions (right panel) to assess the linear and native protein conformations. Molecular weight marker (kDa) was loaded in lane 1. Glycosylated YghJ was loaded in lanes 2 and 4 whereas the non-glycosylated protein variant was loaded in lanes 3 and 5. Chicken anti-FLAG antibodies (diluted x4.000) and HRP conjugated rabbit Anti-Chicken IgY (diluted x5.000) were used to visualize YghJ.

The extensive O-linked protein glycosylation observed in ETEC indicates a role for these modifications in normal cellular physiology in addition to virulence (Boysen et al., 2016; Maigaard Hermansen et al., 2018). We speculated if this abundant but overlooked type of protein modification for example could influence protein conformation. To examine this, we first purified and characterized a glycosylated and non-glycosylated version of YghJ. As for the BEMAP analysis, glycosylated YghJ was isolated from the ETEC culture supernatant. In contrast, the non-glycosylated YghJ version was ectopically expressed, and affinity purified from a commensal *E. coli* MG1655Δ*hldE* mutant background. Pathogenic *E. coli* uses mono heptoses for protein glycosylation and the synthesis of these glycans depends on the *hldE* gene (Benz and Schmidt, 2001; Nakao et al., 2012; Boysen et al., 2016). Therefore, with the deletion of *hldE*, the expression strain would lose its ability to modify YghJ with this type of glycan. To assess the conformation of glycosylated YghJ and the non-glycosylated counterpart, the linear and native structure of both proteins were compared using reducing and native PAGE analysis, respectively. As shown in **Figure 1**, several bands carrying the FLAG-tag were identified for both glycosylated and non-glycosylated YghJ. These bands reflect degradation of the proteins during the purification process. It is observed that both proteins displayed similar migration patterns and the degradation fragments were of equal sizes. Next, we verified the non-modified state of YghJ isolated from the MG1655Δ*hldE* expression strain, using 40 μg purified protein as input to a BEMAP analysis. No modified sites were identified (data not shown). Based on the PAGE analysis and BEMAP, we conclude that our purification approach allows us to isolate glycosylated YghJ as well as a non-glycosylated version. In addition, the modifications do not appear to dramatically influence the overall protein structure, but it is possible that the glycosylation could induce local conformational changes throughout YghJ (Shental-Bechor and Levy, 2008).

### YghJ Glycosylation Is Associated With Increased Recognition by Human Immune Response

The controlled human infection model (CHIM) has advanced the understanding of ETEC pathogenesis, assisted in characterizing the gut mucosal immune system and aided the search for candidate vaccine antigens, as well as evaluating early candidates in Phase 1/2/2B clinical trials (Chakraborty et al., 2016; Chakraborty et al., 2018; Vedøy et al., 2018). In this study we use sera from the CHIM study to investigate the human YghJ-specific immune response following infection (Chakraborty et al., 2015). Only individuals which experienced a moderate to severe diarrhea when ingesting ETEC were included in this analysis.

**Figure 2:**
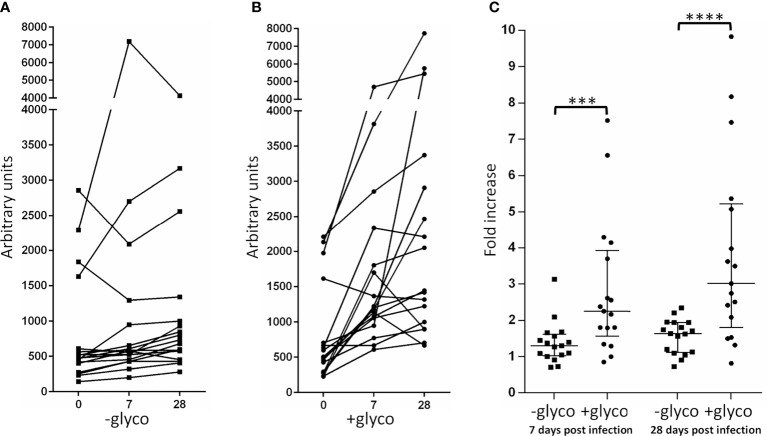
ELISA experiment using 17 serum samples from a controlled human infection model (CHIM) study showing the relative immune response towards glycosylated YghJ (+glyco) and the non-glycosylated protein variant (-glyco). **(A)** Arbitrary anti non-glycosylated YghJ IgG/A/M antibody levels in serum 0, 7 and 28 days after ETEC ingestion is shown. **(B)** Arbitrary anti glycosylated YghJ IgG/A/M antibody levels in serum 0, 7 and 28 days after ETEC ingestion is shown. **(C)** The ratio between measured endpoint titers obtained at Day 0 and Day 7 as well as Day 0 and Day 28 for antibodies that bound –glyco (squares) or +glyco (circles) was calculated and plotted. A Wilcoxon matched-pairs signed rank test was performed to evaluate significant differences in the immune response towards glycosylated and non-glycosylated YghJ. ***P = 0.0003. ****P < 0.0001. Median with interquartile range for each data set is indicated.

As shown above, the 1519 amino acid protein YghJ is glycosylated at 54 different Ser/Thr residues. Therefore, these glycan-peptide epitopes constitute only a fraction of YghJ epitopes presented to the immune system during infection. Nevertheless, we speculated whether the ingestion of ETEC and thus an exposure to glycosylated YghJ would raise an immune response, which differed in recognition compared to the non-glycosylated protein variant. Specifically, we investigated the difference between antibodies recognizing the glycosylated YghJ as compared to the non-glycosylated variant using 17 serum samples from the CHIM study. In order to quantitatively assess the immune response, we conducted ELISA experiments. Serum isolated from the individuals before infection (Day 0), after 7 days (Day 7) and 28 days (Day 28) post infection was used as input for the analysis. The relative serum antibody response towards the glycosylated and non-glycosylated antigen is shown in **Figures 2A, B**. As shown in **Figure 2C**, we have plotted the relative increase in immune response towards the two antigens by comparing the Day 0 samples to either Day 7 or the Day 28 samples in a patient by patient manner. In our analysis, serum samples withdrawn from patients on Day 7 showed a significantly stronger response (p = 0.0003) towards the glycosylated YghJ (+glyco) compared to the non-modified protein variant (-glyco) with calculated medians of 2.3 and 1.3, respectively. The response towards glycosylated YghJ and the non-modified protein variant became even more pronounced on Day 28 (p = 0.0001). The patient-to-patient variability in antibodies recognizing glycosylated YghJ increased and the median rose from 2.3 to 3.0. On the other hand, the level of patient antibodies recognizing non-glycosylated YghJ was more uniformly distributed and the median increased modestly from 1.3 to 1.6.

To ensure that serum ELISA signal depended only on YghJ recognizing antibodies, two control experiments were performed. In one control experiment we used Western blotting and serum samples from three patients to probe for glycosylated YghJ to determine if co-purified contaminants had contributed to the ELISA signals. In this analysis, we detected one specific YghJ signal suggesting our results exclusively are based on antibodies recognizing our antigen (**Supplementary Figures S2** and **S3**). In another control experiment ELISA plates were coated with a nonsense protein and the serum response measured. Only low titers, with no correlation to sample day were observed, supporting that we have measured an YghJ-specific antibody response in our experimental setup (data not shown).

Remarkably, even though the protein glycosylation only constitutes a minor fraction of all the possible epitopes, our ELISA results show that the immune response towards glycosylated YghJ is stronger than that of the non-glycosylated protein variant. These results assign a clear role to protein glycosylation in the immune response against the YghJ antigens, both natural and recombinant.

## Discussion

BEMAP is a sensitive, selective and robust method which enables the identification of O-linked glycosylated sites in proteins (Boysen et al., 2016). When screening for glycosylated proteins in ETEC H10407, four glycosylated YghJ Ser/Thr sites were initially identified within the 1519 amino acid sequence. In this study, we have used BEMAP to further study glycosylated YghJ purified from ETEC H10407. As shown in **Table 1**, our analysis increases the number of identified sites from four to a total of 54 and we conclude that YghJ is hyperglycosylated. The macro heterogeneity, or glycan site occupancy, for each Ser/Thr residue within YghJ remains to be determined but we speculate that the site variation may be just as significant as observed in the Eukaryotic domain (Čaval et al., 2021). The BEMAP method can only determine if a site is modified or not. Therefore, if the site occupancy varies, it is possible that even more sites may be identified if more protein is analyzed and/or the sensitivity of the used MS instrument is increased. Based on the results presented here, we have firmly established that YghJ is hyperglycosylated and our observations indicate that this non-canonical ETEC antigen should be added to the growing list of modified bacterial proteins with potential vaccine importance. Moreover, as demonstrated with YghJ, we highlight that the BEMAP method has the potential to reveal novel insights into proteins already extensively characterized (Luo et al., 2014; Nesta et al., 2014).

Post translational protein modifications in the prokaryotic world have until recently been regarded as rare and exotic. However, increasing efforts are being dedicated to understanding the functions and benefit of protein glycosylation in the context of immunogenicity. Some studies have for example demonstrated that the glycosylation mask the surface of the protein or even forms a glycan-shield in order to avoid recognition by the immune system of the host (Gault et al., 2015; Walls et al., 2016). The mapping of the glycosylated residues onto a Phyre2 based 3D model (Kelley et al., 2015) of YghJ (**Supplementary Figure S1**) reveals extensive surface exposure of the glycans. Therefore one could imagine a similar role for YghJ protein glycosylation. This hypothesis, however, is rejected by our data presented in **Figure 2**, showing that YghJ glycosylation increases the overall immunogenicity of the protein although they only constitute a small fraction of all the available epitopes.

In this study, we have preliminarily investigated if these modifications induce topology changes of YghJ by comparing the native and denatured conformation of the protein. As presented in **Figure 1**, the PAGE analysis did not reveal any detectable differences between glycosylated YghJ and the non-modified protein variant when examined under the different experimental conditions. This indicates, that if the glycosylation does induce conformational changes in YghJ, they are local. However, the data does not exclude the possibility that O-linked glycosylation serves to influence folding kinetics, protein stability or contributes to protein function as seen with other glycoproteins (Knudsen et al., 2008; Shental-Bechor and Levy, 2008).

Despite an increasingly important unmet public health need, there is still a large number of important bacterial pathogens such as ETEC where the first effective vaccine has yet to enter the market. The evidence of widespread bacterial O-linked protein glycosylation and the impact of these modifications on function and immunogenicity is accumulating at an increasing rate. We speculate that the failure to produce safe and high efficacy subunit vaccines targeting bacterial pathogens may in part be associated with absence of immunologically important O-linked glycosylations in the final protein antigen formulation. The absence of these glycosylations could for example arise if protein antigens are produced in an *E. coli* K-12 background. In a previous study we identified cell surface-associated glycoproteins from ETEC and *E. coli* K-12 (Boysen et al., 2016). Here we observed that the majority of the ETEC glycoproteins were conserved in both strains but nevertheless were only glycosylated in the pathogens. This suggests that antigen expression in *E. coli* K-12 will result in little or no protein glycosylation at all. As described, all CHIM patients carried antibodies against YghJ on day 0 and 67% of the enrolled patients became ill when exposed to a dose of ETEC. This suggests that prior exposure to *E. coli* did not raise antibodies against YghJ or any other *E. coli* antigen, associated with cross-protection against ETEC. This lack of protection may be a matter of antibody threshold levels. It has previously been reported, that the higher the baseline level of serum IgA and IgG against ETEC in unvaccinated individuals, the lower the incidence of moderate to severe illness (McKenzie et al., 2008). With this study, using serum from an ETEC controlled human infection study, we have provided an important link between protein glycosylation and the immunogenicity on an established and well investigated non-canonical ETEC antigen. It is possible that YghJ could show the same level of cross-protection as demonstrated in *Shigella* using the conserved protein outer membrane protein PSSP-1 (Kim et al., 2018). Future investigations will include specific epitope analyses of the glycosylated YghJ and the non-modified protein variant to further study the antigenic potential of O-linked glycosylation. We believe that the increased immunogenicity of glycosylated YghJ compared to the non-modified protein variant will prove to be a distinguishing factor impacting on the ability of this protein to induce protective immune responses and thus enhance its potential as a non-canonical antigen component of future diarrheagenic and uropathogenic *E. coli* vaccines.

## Data Availability Statement

The datasets presented in this study can be found in online repositories. The names of the repository/repositories and accession number(s) can be found below: ProteomeXchange, PXD025876.

## Ethics Statement

The studies involving human participants were reviewed and approved by The CHIMs study with H01047 was conducted under BB-IND-12,243 at the Center for Immunization Research (CIR) and the Johns Hopkins Bloomberg School of Public Health (JHBSPH), Baltimore, MD, USA. Ethic approval to conduct the study was provided by the Western Institutional Review Board (WIRB) (Olympia, WA) for JHBSPH and PATH and by the Institutional Biosafety Committee of the Johns Hopkins Medical Institutions. The patients/participants provided their written informed consent to participate in this study.

## Author Contributions

MT, LV, ML, AA and AB conceived and designed the experiments. MT, SC, ALB, AA and AB wrote the manuscript. MT, TK, AN, SC, ALB, AA and AB analyzed the data. MT and RJ purified protein for all experiments. SC and ALB supplied the CHIMs serum samples. MT and RJ performed PAGE experiments. LV and AB performed ELISA. AN, ML and AB performed mass spectrometry analyses. All authors contributed to the article and approved the submitted version.

## Funding

This work was supported, in whole or in part, by the Innovation Fund Denmark grant 7041-00220 and Bill & Melinda Gates Foundation grant OPP1112376. Under the grant conditions of the Foundation, a Creative Commons Attribution 4.0 Generic License has already been assigned to the Author Accepted Manuscript version that might arise from this submission.

## Conflict of Interest

AB and ML are listed as inventors on patent applications relating to WO 2017/059864 related to the glycosylated YghJ protein held by University of Southern Denmark and Aarhus University. AB, ML and AA have a financial interest in GlyProVac ApS, which has licensed exclusively the IP stated above. AB is the scientific founder, shareholder, and a member of the board. AA is co- founder, shareholder, and a member of the board. ML is a shareholder. AB, AA, MT, TK, and RJ are all employees of GlyProVac ApS.

The remaining authors declare that the research was conducted in the absence of any commercial or financial relationships that could be construed as a potential conflict of interest.

## Publisher’s Note

All claims expressed in this article are solely those of the authors and do not necessarily represent those of their affiliated organizations, or those of the publisher, the editors and the reviewers. Any product that may be evaluated in this article, or claim that may be made by its manufacturer, is not guaranteed or endorsed by the publisher.

## References

[B1] Anderson J. D. Bagamian K. H. Muhib F. Amaya M. P. Laytner L. A. Wierzba T.. (2019). Burden of Enterotoxigenic Escherichia Coli and Shigella non-Fatal Diarrhoeal Infections in 79 Low-Income and Lower Middle-Income Countries: A Modelling Analysis. Lancet Glob. Heal. 7 (3), e321–e330. 10.1016/S2214-109X(18)30483-2 PMC6379821 30784633

[B2] Barry E. Cassels F. Riddle M. Walker R. Wierzba T. (2019). Vaccines Against Shigella and Enterotoxigenic Escherichia Coli: A Summary of the 2018 VASE Conference. Vaccine 37 (34), 4768–4774. 10.1016/j.vaccine.2019.02.070 31358236

[B3] Bekeredjian-Ding I. Delany-Moretlwe S. Fritzell B. Kang G. Karron R. Kaslow D.. (2020). WHO Product Development for Vaccines Advisory Committee (PDVAC) Virtual Consultation 4: Update on Development of Enterotoxigenic E.coli (ETEC) Vaccines 18 June 2020. Prod. Dev. Vaccines Advis. Commun. 0 (0), 1–15.

[B4] Benz I. Schmidt M. A. (2001). Glycosylation With Heptose Residues Mediated by the Aah Gene Product is Essential for Adherence of the AIDA-I Adhesin. Mol. Microbiol. 40 (6), 1403–1413. 10.1046/j.1365-2958.2001.02487.x 11442838

[B5] Boysen A. Moller-Jensen J. Kallipolitis B. Valentin-Hansen P. Overgaard M. (2016). A Novel Mass Spectrometric Strategy ‘Bemap’ Reveals Extensive O-Linked Protein Glycosylation in Enterotoxigenic Escherichia Coli. Sci. Rep. 6, 32016. 10.1038/srep32016 27562176 PMC5000012

[B6] Boysen A. Moller-Jensen J. Kallipolitis B. Valentin-Hansen P. Overgaard M. (2010). Translational Regulation of Gene Expression by an Anaerobically Induced Small Non-Coding RNA in Escherichia Coli. J. Biol. Chem. 285 (14), 10690–10702. 10.1074/jbc.M109.089755 20075074 PMC2856277

[B7] Castric P. Cassels F. J. Carlson R. W. (2001). Structural Characterization of the Pseudomonas Aeruginosa 1244 Pilin Glycan. J. Biol. Chem. 276 (28), 26479–26485. 10.1074/jbc.M102685200 11342554

[B8] Čaval T. Heck A. J. R. Reiding K. R. (2021). Meta-Heterogeneity: Evaluating and Describing the Diversity in Glycosylation Between Sites on the Same Glycoprotein. Mol. Cell. Proteomics. 20, 1–14. 10.1074/MCP.R120.002093 PMC8724623 33561609

[B9] Chakraborty S. Brubaker J. Harro C. Weirzba T. Sack D. (2019). Development of a Novel Multiplex Electrochemiluminescent-Based Immunoassay to Aid Enterotoxigenic Escherichia Coli Vaccine Development and Evaluations. J. Immunol. Methods 470, 6–14. 10.1016/j.jim.2019.04.003 31004579 PMC6538825

[B10] Chakraborty S. Brubaker J. Harro C. Weirzba T. Sack D. (2015). Characterization of Mucosal Immune Responses to Enterotoxigenic Escherichia Coli Vaccine Antigens in a Human Challenge Model: Response Profiles After Primary Infection and Homologous Rechallenge With Strain H10407. Clin. Vaccine Immunol. 23 (1), 55–64. 10.1128/CVI.00617-15 26581889 PMC4711095

[B11] Chakraborty S. Harro C. DeNearing B. Ram M. Feller A. Cage A.. (2016). Characterization of Mucosal Immune Responses to Enterotoxigenic Escherichia Coli Vaccine Antigens in a Human Challenge Model: Response Profiles After Primary Infection and Homologous Rechallenge With Strain H10407. Clin. Vaccine Immunol. 23 (1), 55–64. 10.1128/CVI.00617-15 26581889 PMC4711095

[B12] Chakraborty S. Randall A. Vickers T. J. Molina D. Harro C. D. DeNearing B.. (2018). Human Experimental Challenge With Enterotoxigenic Escherichia Coli Elicits Immune Responses to Canonical and Novel Antigens Relevant to Vaccine Development. J. Infect. Dis. 218 (9), 1436–1446. 10.1093/infdis/jiy312 29800314 PMC6151082

[B13] Datsenko K. A. Wanner B. L. (2000). One-Step Inactivation of Chromosomal Genes in Escherichia Coli K-12 Using PCR Products. Proc. Natl. Acad. Sci. U. S. A. 97 (12), 6640–6645. 10.1073/pnas.120163297 10829079 PMC18686

[B14] Faulds-Pain A. Twine S. M. Vinogradov E. Strong P. C. R. Dell A. Buckley A. M.. (2014). The Post-Translational Modification of the Clostridium Difficile Flagellin Affects Motility, Cell Surface Properties and Virulence. Mol. Microbiol. 94 (2), 272–289. 10.1111/mmi.12755 25135277 PMC4441256

[B15] Fleckenstein J. Sheikh A. Qadri F. (2014). Novel Antigens for Enterotoxigenic Escherichia Coli Vaccines. Expert Rev. Vaccines 13 (5), 631–639. 10.1586/14760584.2014.905745 24702311 PMC4199203

[B16] Gault J. Ferber M. Machata S. Imhaus A. F. Malosse C. Charles-Orszag A.. (2015). Neisseria Meningitidis Type IV Pili Composed of Sequence Invariable Pilins Are Masked by Multisite Glycosylation. PLoS Pathog. 11 (9), e1005162. 10.1371/journal.ppat.1005162 26367394 PMC4569582

[B17] Giersing B. (2020). DRAFT WHO Preferred Product Characteristics for Vaccines Against Enterotoxigenic Escherichia Coli no. May

[B18] Hanahan D. (1983). Studies on Transformation of Escherichia Coli With Plasmids. J. Mol. Biol. 166, 557–580. 10.1016/S0022-2836(83)80284-8 6345791

[B19] Iwashkiw J. A. Seper A. Weber B. S. Scott N. E. Vinogradov E. Stratilo C.. (2012). Identification of a General O-Linked Protein Glycosylation System in Acinetobacter Baumannii and its Role in Virulence and Biofilm Formation. PLoS Pathog. 8 (6), e1002758. 10.1371/journal.ppat.1002758 22685409 PMC3369928

[B20] Kansal R. Rasko D. A. Sahl J. W. Munson G. P. Roy K. Luo Q.. (2013). Transcriptional Modulation of Enterotoxigenic Escherichia Coli Virulence Genes in Response to Epithelial Cell Interactions. Infect. Immun. 81 (1), 259–270. 10.1128/IAI.00919-12 23115039 PMC3536156

[B21] Kelley L. A. Mezulis S. Yates C. M. Wass M. N. Sternberg M. J. (2015). The Phyre2 Web Portal for Protein Modeling, Prediction and Analysis. Nat. Protoc. 10, 845–858. 10.1038/nprot.2015-053 25950237 PMC5298202

[B22] Khalil I. Walker R. Porter C. K. Muhib F. Chilengi R. Cravioto A.. (2021). Enterotoxigenic Escherichia Coli (ETEC) Vaccines: Priority Activities to Enable Product Development, Licensure, and Global Access. Vaccinexxxx. 10.1016/j.vaccine.2021.04.018 PMC8273896 33965254

[B23] Kim M. J. Moon Y. H. Kim H. J. Rho S. Shin Y. K. Song M.. (2018). Cross-Protectiveshigella Whole-Cell Vaccine With a Truncated O-Polysaccharide Chain. Front. Microbiol. 9, 2609. 10.3389/fmicb.2018.02609 30429838 PMC6220597

[B24] Knudsen S. K. Stensballe A. Franzmann M. Westergaard U. B. Otzen D. E. (2008). Effect of Glycosylation on the Extracellular Domain of the Ag43 Bacterial Autotransporter: Enhanced Stability and Reduced Cellular Aggregation. Biochem. J. 412 (3), 563–577. 10.1042/BJ20071497 18341480

[B25] Lithgow K. V. Scott N. E. Iwashkiw J. A. Thomson E. L. Foster L. J. Feldman M. F.. (2014). A General Protein O-Glycosylation System Within the Burkholderia Cepacia Complex is Involved in Motility and Virulence. Mol. Microbiol. 92 (1), 116–137. 10.1111/mmi.12540 24673753

[B26] Luo Q. Kumar P. Vickers T. J. Sheikh A. Lewis W. G. Rasko D. A.. (2014). Enterotoxigenic Escherichia Coli Secretes a Highly Conserved Mucin-Degrading Metalloprotease to Effectively Engage Intestinal Epithelial Cells. Infect. Immun. 82 (2), 509–521. 10.1128/IAI.01106-13 24478067 PMC3911403

[B27] Maigaard Hermansen G. M. Boysen A. Krogh T. J. Nawrocki A. Jelsbak L. Moller-Jensen J. (2018). HldE Is Important for Virulence Phenotypes in Enterotoxigenic Escherichia Coli. Front. Cell Infect. Microbiol. 8, 253. 10.3389/fcimb.2018.00253 30131942 PMC6090259

[B28] McKenzie R. Darsley M. Thomas N. Randall R. Carpenter C. Forbes E.. (2008). A Double-Blind, Placebo-Controlled Trial to Evaluate the Efficacy of PTL-003, an Attenuated Enterotoxigenic E. Coli (ETEC) Vaccine Strain, in Protecting Against Challenge With Virulent ETEC. Vaccine 26 (36), 4731–4739. 10.1016/j.vaccine.2008.06.064 18602960

[B29] Mirhoseini A. Amani J. Nazarian S. (2018). Review on Pathogenicity Mechanism of Enterotoxigenic Escherichia Coli and Vaccines Against it. Microb. Pathog. 117, 162–169. 10.1016/j.micpath.2018.02.032 29474827

[B30] Mohamed Y. F. Scott N. E. Molinaro A. Creuzenet C. Ortega X. Lertmemongkolchai G.. (2019). A General Protein O-Glycosylation Machinery Conserved in Burkholderia Species Improves Bacterial Fitness and Elicits Glycan Immunogenicity in Humans. J. Biol. Chem. 294 (36), 13248–13268. 10.1074/jbc.RA119.009671 31350337 PMC6737235

[B31] Moriel D. G. Bertoldi I. Spagnuolo A. Marchi S. Rosini R. Nesta B.. (2010). Identification of Protective and Broadly Conserved Vaccine Antigens From the Genome of Extraintestinal Pathogenic Escherichia Coli. Proc. Natl. Acad. Sci. U. S. A. 107 (20), 9072–9077. 10.1073/pnas.0915077107 20439758 PMC2889118

[B32] Moriel D. G. Rosini R. Seib K. L. Serino L. Pizza M. Rappuoli R. (2012). Escherichia Coli: Great Diversity Around a Common Core. MBio 3 (3), 6–8. 10.1128/mBio.00118-12 PMC3374390 22669628

[B33] Nakao R. Ramstedt M. Wai S. N. Uhlin B. E. (2012). Enhanced Biofilm Formation by Escherichia Coli LPS Mutants Defective in Hep Biosynthesis. PLoS One 7 (12), e51241. 10.1371/journal.pone.0051241 23284671 PMC3532297

[B34] Nesta B. Valeri M. Spagnuolo A. Rosini R. Mora M. Donato P.. (2014). SslE Elicits Functional Antibodies That Impair In Vitro Mucinase Activity and In Vivo Colonization by Both Intestinal and Extraintestinal Escherichia Coli Strains. PloS Pathog. 10, 1–12. 10.1371/journal.ppat.1004124 PMC4014459 24809621

[B35] Nesta B. Pizza M. (2018). Vaccines Against Escherichia Coli. Curr. Top. Microbiol. Immunol. 416, 213–242. 10.1007/82_2018_111 30062594

[B36] Olson S. Hall A. Riddle M. S. Porter C. K. (2019). Travelers’ Diarrhea: Update on the Incidence, Etiology and Risk in Military and Similar Populations - 1990-2005 Versus 2005-2015, Does a Decade Make a Difference? Trop. Dis. Travel Med. Vaccines 5 (1), 1–15. 10.1186/s40794-018-0077-1 30675367 PMC6332902

[B37] Perez-Riverol Y. Csordas A. Bai J. Bernal-Llinares M. Hewapathirana S. Kundu D. J.. (2019). The PRIDE Database and Related Tools and Resources in 2019: Improving Support for Quantification Data. Nucleic Acids Res 47, D442–D450. 10.1093/nar/gky1106 30395289 PMC6323896

[B38] Poolman J. T. Anderson A. S. (2018). Escherichia Coli and Staphylococcus Aureus: Leading Bacterial Pathogens of Healthcare Associated Infections and Bacteremia in Older-Age Populations. Expert Rev. Vaccines 17 (7), 607–618. 10.1080/14760584.2018.1488590 29902092

[B39] Roy K. Bartels S. Qadri F. Fleckenstein J. M. (2010). Enterotoxigenic Escherichia Coli Elicits Immune Responses to Multiple Surface Proteins. Infect. Immun. 78 (7), 3027–3035. 10.1128/IAI.00264-10 20457787 PMC2897383

[B40] Sambrook J. Green M. R. (2012). Molecular Cloning: A Laboratory Manual. 4th ed Vol. 3 (New York: Cold Spring Harbor Laboratory Press).

[B41] Schäffer C. Messner P. (2017). Emerging Facets of Prokaryotic Glycosylation. FEMS Microbiol. Rev. 41 (1), 49–91. 10.1093/femsre/fuw036 27566466 PMC5266552

[B42] Scott N. E. (2019). Expanding Our Understanding of the Role of Microbial Glycoproteomes Through High-Throughput Mass Spectrometry Approaches. Glycoconj. J. 36, 259–266. 10.1007/s10719-019-09875-1 31270739

[B43] Serino L. Pizza M. Gomes Moriel D. Fontana Maria R. (2010). Escherichia Coli Immunogens With Improved Solubility. 1–29. WO 2009/104092.

[B44] Shental-Bechor D. Levy Y. (2008). Effect of Glycosylation on Protein Folding: A Close Look at Thermodynamic Stabilization. Proc. Natl. Acad. Sci. U. S. A. 105 (24), 8256–8261. 10.1073/pnas.0801340105 18550810 PMC2448824

[B45] Shrivastava S. R. Shrivastava P. S. Ramasamy J. (2018). World Health Organization Releases Global Priority List of Antibiotic-Resistant Bacteria to Guide Research, Discovery, and Development of New Antibiotics. JMS J. Med. Soc 32 (1), 76–77. 10.4103/jms.jms_25_17

[B46] Tacconelli E. Carrara E. Savoldi A. Harbarth S. Mendelson M. Monnet D. L.. (2018). Discovery, Research, and Development of New Antibiotics: The WHO Priority List of Antibiotic-Resistant Bacteria and Tuberculosis. Lancet Infect. Dis. 18 (3), 318–327. 10.1016/S1473-3099(17)30753-3 29276051

[B47] Tapader R. Bose D. Pal A. (2017). YghJ, the Secreted Metalloprotease of Pathogenic E. Coli Induces Hemorrhagic Fluid Accumulation in Mouse Ileal Loop. Microb. Pathog. 105, 96–99. 10.1016/j.micpath.2017.02.020 28212863

[B48] Tapader R. Bose D. Basu P. Mondal M. Mondal A. Chatterjee N. S.. (2016). Role in Proinflammatory Response of YghJ, a Secreted Metalloprotease From Neonatal Septicemic Escherichia Coli. Int. J. Med. Microbiol. 306 (7), 554–565. 10.1016/j.ijmm.2016.06.003 27389679

[B49] Theuretzbacher U. Outterson K. Engel A. Karlén A. (2019). The Global Preclinical Antibacterial Pipeline. Nat. Rev. Microbiol. 2019, 1–11. 10.1038/s41579-019-0288-0 PMC7223541 31745331

[B50] Turner S. M. Chaudhuri R. R. Jiang Z. D. DuPont H. Gyles C. Penn C. W.. (2006). Phylogenetic Comparisons Reveal Multiple Acquisitions of the Toxin Genes by Enterotoxigenic Escherichia Coli Strains of Different Evolutionary Lineages. J. Clin. Microbiol. 44 (12), 4528–4536. 10.1128/JCM.01474-06 17050815 PMC1698409

[B51] Uzzau S. Figueroa-Bossi N. Rubino S. Bossi L. (2001). Epitope Tagging of Chromosomal Genes in Salmonella. Proc. Natl. Acad. Sci. U.S.A. 98 (26), 15264–15269. 10.1073/pnas.261348198 11742086 PMC65018

[B52] Vedøy O. B. Hanevik K. Sakkestad S. T. Sommerfelt H. Steinsland H. (2018). Proliferation of Enterotoxigenic Escherichia Coli Strain TW11681 in Stools of Experimentally Infected Human Volunteers. Gut Pathog. 10 (1), 1–8. 10.1186/s13099-018-0273-6 30349586 PMC6192177

[B53] Vik Å. Aas F. E. Anonsen J. H. Bilsborough S. Schneider A. Egge-Jacobsen W.. (2009). Broad Spectrum O-Linked Protein Glycosylation in the Human Pathogen Neisseria Gonorrhoeae. Proc. Natl. Acad. Sci. U. S. A. 106 (11), 4447–4452. 10.1073/pnas.0809504106 19251655 PMC2648892

[B54] Walls A. C. Tortorici M. A. Frenz B. Snijder J. Li W. Rey F. A.. (2016). Glycan Shield and Epitope Masking of a Coronavirus Spike Protein Observed by Cryo-Electron Microscopy. Nat. Struct. Mol. Biol. 10.1038/nsmb.3293 PMC5515730 27617430

[B55] Wellcome Trust. (2019). Vaccines to Tackle Drug Resistant Infections An Evaluation of R&D Opportunities.

[B56] World Health Organization. (2020). Leveraging Vaccines to Reduce Antibiotic Use and Prevent Antimicrobial Resistance: An Action Framework. 10.1093/cid/ciab062 PMC8366823 33493317

