# Supplementary material for: Linking inherent O-Linked Protein Glycosylation of YghJ to Increased Antigen Potential
Source: Front Cell Infect Microbiol. 2021 Aug 19;11:705468. doi: 10.3389/fcimb.2021.705468 (PMC8417355; doi:10.3389/fcimb.2021.705468)
Supplement: Supplementary file 1 [file Image_1.pdf]

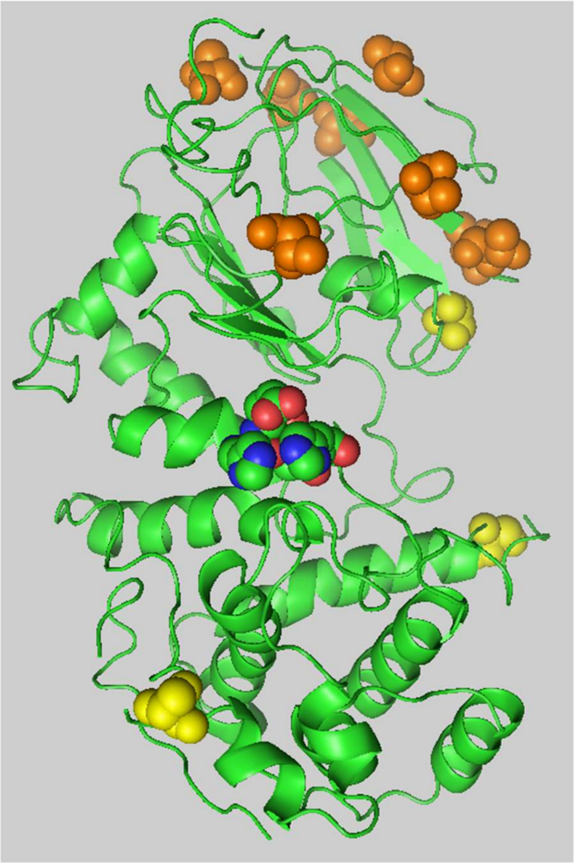

Supplementary figure S1. Protein model as predicted by Phyre2. Only the last 500 amino acids of the C-terminal structure could be predicted. Yellow and orange spheres indicate Ser and Thr glycosylation identified using BEMAP, respectively. Blue and red spheres indicate the catalytic site of YghJ (The Phyre2 web portal for protein modeling, prediction and analysis. Kelley LA et al. Nature Protocols 10, 845-858 (2015)).
