# Supplementary material for: Linking inherent O-Linked Protein Glycosylation of YghJ to Increased Antigen Potential
Source: Front Cell Infect Microbiol. 2021 Aug 19;11:705468. doi: 10.3389/fcimb.2021.705468 (PMC8417355; doi:10.3389/fcimb.2021.705468)
Supplement: Supplementary file 2 [file Image_2.pdf]

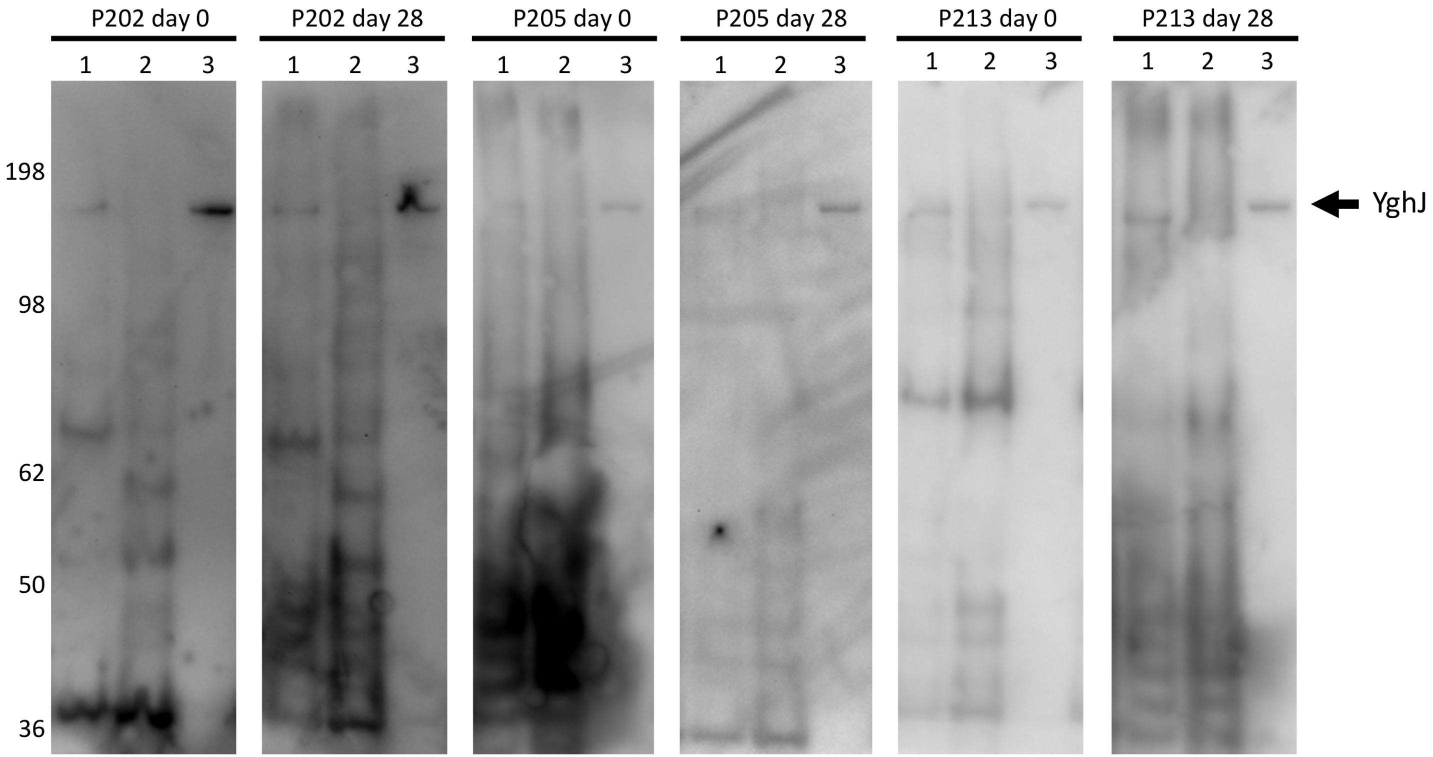

Supplementary Figure S2: Control Western blot showing only purified YghJ is being recognized by antibodies in CHIM patient serum. Culture supernatant from ETEC H10407 (lane 1), an isogenic ETEC H10407Δ*yghJ* knockout mutant (lane 2) or 100ng purified glycosylated YghJ (lane 3) was loaded onto a PAGE gel and run under reducing conditions. Molecular weight marker (kDa) is shown to the left. Anonymized patient serum withdrawn at day 0 and 28 after ETEC ingestion from three individuals was used as primary antibody (diluted x1.000). Secondary IgG/A/M HRP (DAKO P0212) was diluted x8.000. Sample P202, P205 and P213 refer to the anonymized patient number.
