# Supplementary material for: Linking inherent O-Linked Protein Glycosylation of YghJ to Increased Antigen Potential
Source: Front Cell Infect Microbiol. 2021 Aug 19;11:705468. doi: 10.3389/fcimb.2021.705468 (PMC8417355; doi:10.3389/fcimb.2021.705468)

Supplementary Figure S3: Coomassie staining and Western blot analysis of purified glycosylated and non-glycosylated YghJ as well as mouse α-FLAG IgG antibody. Left panel: 0.2ug purified glycosylated and non-glycosylated YghJ as well as 0.4ug mouse α-FLAG IgG antibody was loaded onto a PAGE gel and run under reducing conditions. Proteins were visualized using Coomassie blue (Candiano et al. Electrophoresis, 2004). Molecular weight marker (kDa) is indicated. Right panel: 0.2ug purified glycosylated and non-glycosylated YghJ as well as 0.4ug mouse α-FLAG IgG antibody was loaded onto a PAGE gel and run under reducing conditions. Western blot was used to visualize proteins. Primary mouse α-FLAG IgG antibody (diluted x50.000). Secondary mouse IgG was diluted x8.000. Molecular weight marker (kDa) is indicated.

The Coomassie stained gel (left panel) in figure shows YghJ in the expected mass range and the same extent of degradation for both glycosylated and non-glycosylated YghJ (also shown in Figure 1). In the Western blot (right panel), some of the extra bands below YghJ can be observed. These bands can be assigned to monoclonal mouse α-FLAG IgG which bleeds from the resin during purification protocol. The Coomassie stained gel also shows that no contaminating protein(s) can be observed in the mass range below what the Western Blot in Supplementary Figure S2 reveals i.e., 3-35 kDa.

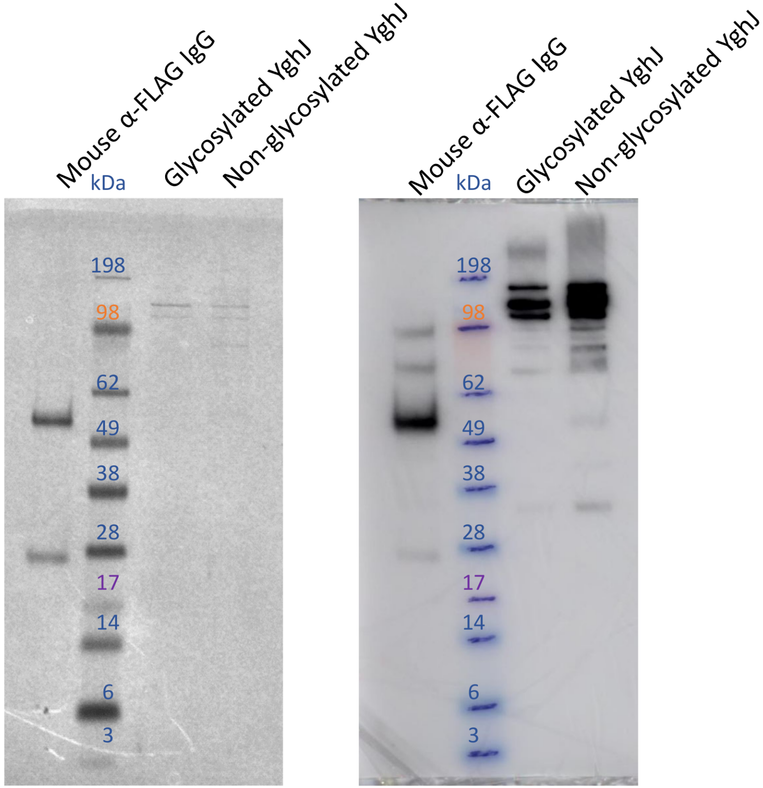

Supplement: Supplementary file 3 [file Image_3.pdf]
