# Supplementary material for: Linking inherent O-Linked Protein Glycosylation of YghJ to Increased Antigen Potential
Source: Front Cell Infect Microbiol. 2021 Aug 19;11:705468. doi: 10.3389/fcimb.2021.705468 (PMC8417355; doi:10.3389/fcimb.2021.705468)
Supplement: Supplementary file 4 [file Table_1.docx]

Supplementary table S1: Strains, plasmids and cell lines used in this study.

| Strains | Genotype | Antibiotic resistance | Reference |
| --- | --- | --- | --- |
| H10407 | ETEC serotype 078:H11; CFA/I, LT/ST |  | (Evans *et al.*, 1975) |
| *E. coli* Top10 | F- *mcrA* Δ(*mrr-hsd*RMS*-mcrBC*) φ80*lac*ZΔM15 Δ*lac*X74 *nup*G *rec*A1 *ara*D139 Δ(*ara-leu*)7697 *gal*E15 *gal*K16 *rps*L(StrR) *end*A1 λ-.  Used for cloning |  | Invitrogen |
| MG1655Δ*hldE* | *hldE* isogenic knockout strain |  | This study |
| H10407*yghJ* | 3xFLAG epitope tagging of yghJ on chromosome |  | This study |
|  |  |  |  |
|  |  |  |  |
| Plasmids |  |  |  |
| pKD46 | Red recombinase expression vector. Temperature sensitive – replicates at 30C | Amp^R^ | (Datsenko & Wanner, 2000) |
| pSUB11 | Used for 3xFLAG epitope tagging on chromosome | Kan^R^ | (Uzzau *et al.*, 2001) |
| pKD3 | Plasmid used for the amplification of the *kan* cassette. | Cml^R^ | (Datsenko & Wanner, 2000) |
| pCP20 | Plasmid bearing the *flp* recombinase gene. Temperature sensitive – replicates at 30C | Amp^R^ | (Datsenko & Wanner, 2000) |
| pXG-0 | Low copy number plasmid | Cml^R^ | (Urban & Vogel, 2007) |
| pGPV104 | Plasmid used for overexpression of ETEC H10407 *yghJ* 3xFLAG | Cml^R^ | This study |

Supplementary table S2: Primers used in this study.

| Primers | Sequence | comment |
| --- | --- | --- |
| JMJ99 | TTATACGCAAGGCGACAAGG | Wanner C1 |
| JMJ388 | TGGTGGAAGAATGAAGTATGGTATTATCGCGCGCAAATTTTGAATCTCTCAGGAGACAGGAATGAAAGTAgtgtaggctggagctgcttcg | hldE KO fw |
| JMJ389 | CAGCGTCAATAGGCCTGCCATGTACGAAGCGAGATCTGTGAACCGCTTTCCGGTTAGCCTTTTTTATCCTGcatatgaatatcctccttagttcc | hldE KO rv |
| JMJ390 | tacgctcatgaaaaaccgaagtta | hldE up |
| JMJ391 | tatatctgcgttccgctaaaaggt | hldE down |
| GPV 16 | TCGTAAATCAATAAAGCCGGATG | yghJ fw |
| GPV 17 | AGCAGCGGAATATTGTCACGTAT | yghJ rv |
| GPV 18 | AAACCGGAACAGGGACCGGAAACCATTAATCAGGTTACCGAGCATAAGATGTCTGCCGAG*GACTACAAAGACCATGACGG* | yghJ 3xFLAG fw |
| GPV 19 | AACCCGGTGCGCCTTATTTCATGCCGGATGCGGCGCGAGCGCCTTATCCGGCCTACGGGC *CATATGAATATCCTCCTTAG* | yghJ 3xFLAG rv |
| GPV 67 | GAAGGAATGGGCAGAGAAAAACT | yghJ fw seg. 7 |
| GPV 95 | ACTTAGATTCAATTGTGAGCCACCATAAGGAGTTTTATAAatgAATAAGAAATTTAAATATAAGA | IPTG SD yghJ fw1 |
| GPV 96 | TAGCTA*CTCGAG*GGCAAAAAGAGTGTTGACTTGTGAGCGGATAACAATGATACTTAGATTCAATTGTGAGCCACCAT | IPTG SD yghJ fw2 |
| GPV 97 | TAGCTAGC TCTAGA TTACTATTTATCGTCGTCATCTTTG | FLAG rv |
| GPV147 | CAGTCATAGCCGAATAGCCT | K1 oligo, Wanner |

Supplementary table S3: Antibodies used in this study.

| Antibody | Dilution factor | Reference |
| --- | --- | --- |
| Polyclonal Rabbit anti-Human IgA, IgG, IgM, Kappa, Lambda-HRP | 1:1000 | Dako P0212 |
| Rabbit Anti-Chicken IgY H&L (HRP) | 1:4000 | Abcam ab6753 |
| Anti-Chicken IgY flag | 1:4000 | Abcam ab1170 |

Datsenko, K.A., and Wanner, B.L. (2000) One-step inactivation of chromosomal genes in Escherichia coli K-12 using PCR products. *Proceedings of the National Academy of Sciences of the United States of America* **97**: 6640-6645.

Evans, D.G., Silver, R.P., Evans, D.J., Jr., Chase, D.G., and Gorbach, S.L. (1975) Plasmid-controlled colonization factor associated with virulence in Esherichia coli enterotoxigenic for humans. *Infect Immun* **12**: 656-667.

Urban, J.H., and Vogel, J. (2007) Translational control and target recognition by Escherichia coli small RNAs in vivo. *Nucleic Acids Res* **35**: 1018-1037.

Uzzau, S., Figueroa-Bossi, N., Rubino, S., and Bossi, L. (2001) Epitope tagging of chromosomal genes in Salmonella. *Proc Natl Acad Sci U S A* **98**: 15264-15269.
